# Extracting metal ions from basic oxygen steelmaking dust by using bio-hydrometallurgy

**DOI:** 10.1016/j.heliyon.2024.e32437

**Published:** 2024-06-04

**Authors:** Ipek Tezyapar Kara, Victoria E. Huntington, Nuannat Simmons, Stuart T. Wagland, Frederic Coulon

**Affiliations:** Cranfield University, School of Water, Energy and Environment, Cranfield, MK430AL, UK

**Keywords:** Bioleaching, Deep eutectic solvent, Metallurgical waste, Metal extraction, Biomining

## Abstract

This study aimed to optimise metal extraction from secondary hazardous sources, such as basic oxygen steelmaking dust (BOS-D). Initially, three batch systems approaches, including bioleaching using *Acidithiobacillus ferrooxidans*, chemical leaching using choline chloride-ethylene glycol (ChCl-EG) and a combined approach were compared. Then, scaling up was evaluated through a semi-continuous bioleaching column system with varied leachate recirculation over 21 days, focusing on Y, Ce, Nd, Li, Co, Cu, Zn, Mn, and Al. Bioleaching outperformed the control experiments within 3 days in the batch, demonstrating the key role of *A. ferrooxidans*. Chemical leaching conducted with a solid concentration of 12.5 % (w/v) successfully dissolved over 50 % of all metals within 2 h. For rare earth elements (REE), both bioleaching and hybrid leaching outperformed chemical leaching. However, considering factors such as process duration, overall efficiency, and ease of extraction, chemical leaching was the most effective method. Leachate recirculation reached a plateau after 11 days, resulting in extraction efficiency of 39 % when semi-continuous column set-up was used. Interestingly, variations in recirculation rates did not influence the extraction efficiency. Overall, this study emphasizes the considerable potential of bioleaching for metal recovery, but also highlights the need for further studies for enhancing permeability for percolation methods and optimisation, particularly in parameters such as aeration rate, when transitioning to larger scale systems.

## Highlights

• Extraction of Y, Ce, Nd, Li, Co, Cu, Zn, Mn, Al from BOS-D was investigated.
• ChCl-EG leaching, bioleaching and a combined approach were evaluated in batch.
• Bioleaching batch achieved higher extraction than control in the first 3 days.
• ChCl-EG leaching was the most efficient for overall metal extraction.
• During column bioleaching, metal extraction reached between 30 % and 70 %.

## 1 Introduction

The steel industry yields various by-products, encompassing slags, dusts, and sludges originating from blast furnaces, basic oxygen furnaces, electric arc furnaces, and sinter plants, along with oily mill scale and pickling sludge [1]. Most of the produced slag is either recycled within the plant or repurposed for different applications such as road construction, fertilizer, and cement components [2]. However, it is worth noting that there are over 190 million tonnes of historical iron and steel slag distributed throughout current and former iron and steel manufacturing regions in the UK [3]. On another note, although basic oxygen steelmaking dust (BOS-D) holds potential as a secondary iron resource owing to its high iron content (40–60 %), it is typically stockpiled on marginal areas due to limitations in its reuse, primarily because of its high zinc content (>1 %) [1,4] Due to zinc low boiling point (907 °C), it vaporizes when introduced into a blast furnace at high temperatures, typically ranging between 1600 and 1650 °C. Zinc (Zn) condenses and accumulates on blast furnace walls that affect the solid and gas flows within the furnace. This leads to decreased productivity and damage the furnace linings [4,5]. Zinc also accelerates the degradation of the furnace's upper stack refractories, further shortening the furnace's operational lifespan [1]. BOS-D also contains wide range of metals including manganese (Mn) and aluminium (Al), as well as critical metals like lithium (Li), cobalt (Co), copper (Cu) and rare earth elements (REEs) such as yttrium (Y), cerium (Ce) and neodymium (Nd). Globally, it is estimated that 20 million tonnes of BOS-D were generated in 2013 [6]. The concept of zero-waste valorisation and the move forward to the circular economy principles highlight the importance of recovering resources from industrial process wastes using cost-effective, sustainable methods like bioleaching [7,8]. This drives researchers to explore technologies that could transform BOS-D into a valuable resource rather than considering it as waste [6,8,9].

To extract metals from complex metal-bearing materials hydrometallurgical methods can provide some advantages over pyrometallurgy due to pyrometallurgical methods require a high capital cost and cause harmful emissions and metal loss during the process [10]. In hydrometallurgy the extraction can be performed ambient to 300 °C and above 100 °C reactions carried out under pressure [11]. Bioleaching, one of the biohydrometallurgical methods, emerges as more environmentally friendly extraction method due to it is mediated by naturally available microorganisms, required low capital cost, and performed under atmospheric pressure [10,12]. Process is influenced by various process parameters such as solid concentration (also termed as pulp density), pH, energy source concentration, temperature, inoculum concentration, agitation speed and recirculation/irrigation. Simultaneous optimisation of these parameters can provide higher metal extraction yield [13,14]. In addition, although scaling up bioleaching brings challenges, amplifying the process will widen the possibilities of implementing the extraction of values on commercial scale as well as will unlock the difficulties and possible solutions by providing technical know-how [9,12,15].

Moreover, the use of green solvents, which derived from agricultural processing, can provide an environmentally friendly alternative to traditional chemicals e.g., EDTA [[16], [17], [18]]. Deep eutectic solvents (DES) are promising discoveries in green chemistry that can be infinitely reused, biodegradable, and non-toxic. The use of green solvents has been proven to recover high yields of critical metals from contaminated waste. Abbott et al. (2009) [16] used choline chloride – urea and ChCl-EG as a deep eutectic solvent and achieved >70 % zinc recovery from electric arc furnace dust. Tran et al. (2019) [18] used ChCl-EG as a deep eutectic solvent on spent lithium-ion batteries and achieved >90 % of lithium and cobalt recovery. Although there are some research that using hybrid leaching process combining chemical leaching with bioleaching to enhance metal extraction form metal-bearing wastes especially from waste electric and electronic equipment, authors have not seen any study using deep eutectic solvent leaching followed by bioleaching for metal extraction from the focused metallurgical waste which is BOS-D [[19], [20], [21]]. Therefore, hybrid leaching processes can potentially enhance metal extractions from BOS-D.

For this study, leachability of metal ions of Y, Ce, Nd, Li, Co, Cu, Zn, Mn, Al from BOS-D was investigated using bio-hydrometallurgy. Bioleaching was evaluated on a semi-continuous column set-up by recirculating the leachate to understand the effect of cycling rate. Also, the efficiency of a hybrid leaching process using ChCl-EG followed by bioleaching was evaluated. This research is important as it provides insights into wider bioleaching applications for secondary resources and sheds light on the limitations and opportunities for scaling up.

## 2 Materials and methods

### 2.1 Sample characterisation and pre-treatment

Bulk BOS-D from a stockpile of a historical iron and steelwork plant(Teesside, UK) was received in a large, sealed drum and stored at ambient temperature. It was air-dried, grounded, and sieved using a 2 mm mesh. The dry matter and water content, and loss on ignition were analysed as described by Tezyapar Kara et al. (2022) [47]. For the bioleaching study, autoclaved BOS-D, for 15 min at 121 °C, was used to ensure precise evaluation of the targeted microbial activity and metal leaching without interference from the native microorganisms [22,23]. The elemental composition of the autoclaved and non-autoclaved materials was determined using aqua regia digestion, with 1:3 ratio of nitric acid (HNO_3_) and hydrochloric acid (HCl), followed by microwave digestion as described by Gutiérrez-Gutiérrez et al. (2015) [24]. Extracts were analysed with a PerkinElmer NexION® Inductively Coupled Plasma Mass spectrometer (ICP-MS) using uranium (U) as internal standard [26]. Single ICP-MS standard solutions sourced from PerkinElmer for calibration were used with the analyte concentration of 10 μg/ml.

### 2.2 Microorganisms and culture conditions

*Acidithiobacillus ferrooxidans* (DSM 583) was sourced from Leibniz Institute (DSMZ), Braunschweig (Germany). *A. ferrooxidans* is a gram-negative, non-sporulating and rod-shaped bacteria that generates leaching agents such as ferric iron (Fe^3+^) and sulphuric acid (H_2_SO_4_) by oxidising ferrous iron (Fe^2+^) and/or reduced sulphur compounds (S_8_, S_2_O_3_^2−^, H_2_S) [12,26,52,53]. The resulting ferric and protons (from sulphuric acid) solubilise the metals from ores/minerals/secondary solid wastes [54]. In this study, FeSO_4_.7H_2_O was used a source of Fe^2+^. *A. ferrooxidans* culture was adapted to 5 % (w/v) of BOS-D as previously described in Tezyapar Kara et al. (2022) [47]. Adapted culture was preserved in Erlenmeyer flasks with a concentration of 10 % (v/v). The culture was maintained in 90 ml of optimised 4.5 K salt medium at pH 1.75 at 30 °C at 150 rpm as described by Chen et al. (2015) [25] and Tezyapar Kara et al. (2023) [26]. Prior to their use in the bioleaching experiment, the adapted cultures were sequentially cultivated for 2 days on two separate occasions. Following the 2 days of cultivation, the pH of the cultures was above 2.00, and the oxidation-reduction potential (ORP) values were ≥600 mV which is a good indicator of a bacterial growth [[27], [28]]. The adapted cultures were then utilised in 250 ml batch optimisation experiments.d A platinum electrode with an Ag/AgCl reference electrode was used for measurements of the redox potential [29]. To prepare the growth medium, a modified salt medium [25] was used. The optimised growth medium consists of two solutions: solution A (modified basal salt medium) and solution B (ferrous iron). The solution A was prepared by adding 2.00 g of (NH_4_)_2_SO_4_, 0.25 g of K_2_HPO_4_, 0.25 g of MgSO_4_·7H_2_O, 0.10 g of KCl and 0.01 g of Ca(NO_3_)_2_ into a 700 ml of deionised water. The solution B was prepared by adding 11.1 g of FeSO_4_.7H_2_O as a source of Fe^2+^, in 300 ml deionised water get 2.22 g/L Fe^2+^ in the medium, respectively. Both solutions were adjusted at pH 2 using 5 M H_2_SO_4_. After pH adjustment, solution A was autoclaved at 121 °C for 15 min. The ferrous iron solution was filtered using 0.2 μm Millipore filter. The solutions A and B were then mixed (7:3). The pH is then adjusted to desired level for bioleaching study using 5 M H_2_SO_4_.

### 2.3 Bioleaching vs chemical leaching vs hybrid leaching

Bioleaching batches were conducted with 1 % (w/v) solid concentration, 1 % (w/v) energy source concentration, 1 % (w/v) inoculum concentration and pH 1.75 as described by Tezyapar Kara et al. (2023) [26]. Bioleaching efficiency was evaluated for nine elements, including Y, Ce, Nd, Li, Co, Cu, Zn, Mn and Al, being the most abundant metals, critical and rare earth elements contained within BOS-D. A two-step bioleaching approach, following the methodology described by Amiri et al. (2011) [30] and Muddanna and Baral (2021) [27] was used. Briefly, BOS-D was introduced to the leaching media after 2 days of *A. ferrooxidans* incubation in the media and this was referred to as a two-step bioleaching process. While Muddanna and Barral (2021) [27] introduce the metal-bearing material in the leaching media after 24 h of *A. ferrooxidans* incubation. ORP was monitored, and ferrous iron concentration was measured as an indicator of bacterial growth on days 0, 2, 3, 5, 8, 11, 16. The pH was adjusted to 1.75 on the same days if needed. The abiotic controls were performed as the inoculated bioleaching batches with 1 % solid concentration using 1 % energy source at pH 1.75. The acid control batches were conducted under identical conditions, albeit with the sole use of 5 M H_2_SO_4_ instead of FeSO_4_.7H_2_O.

The hybrid leaching was performed as follows: initially, a 2-h chemical leaching was conducted using a 1:1 ChCl-EG solution with 12.5 % solid concentration and pH 4. The 100 ml batches were placed in an agitated incubator at room temperature and operating at 150 rpm. The resulting BOS-D residue was then air dried and autoclaved at 121 °C for 15 min before to be used for the subsequent bioleaching phase, which followed the conditions described above. All leaching conditions were performed in duplicate.

### 2.4 Scale-up bioleaching

Column bioleaching was performed to evaluate how the leachate recirculation rate, set at 40 ml/min and 80 ml/min, influenced the metal extraction yield. The dimensions of the PVC columns are 5 cm diameter × 30 cm height. A total of 10 g, equivalent to 1 % (w/v) of air-dried and autoclaved BOS-D was carefully inserted between two layers of glass wool in the column that featured a PVC support plate with multiple holes at its base. The glass wool layers also served as a barrier to prevent suspended solids from entering the reservoir [31,32]. Air flow was supplied to the columns and reservoirs from the bottom at a rate of 1.33 L/min using an air pump (Pawfly®). Continuous leachate circulation of system (40 or 80 ml/min) were supplied using and a peristaltic pump (Watson Marlow 505s). As per the batch bioleaching approach, a two-step approach was used for the bioleaching column system [27,30]. Similar to the batch bioleaching, initially, a growth medium (ORP = 293 mV) of 1 L, containing 1.11 % FeSO_4_·7H_2_O and adjusted to a pH of 2 was inoculated with 1 % (v/v) of a *A. ferrooxidans* culture (ORP ≥600 mV, day 0). Culture was cultivated in the aerated reservoir in a water bath at 30 °C. After 2 days of incubation, when ferrous iron was rapidly consumed compared to abiotic control, rust colour occurred in the leaching medium and the ORP increased the first step was considered complete. For the second step, to initiate the bioleaching, the aerated columns were supplied with the culture from the bottom via the reservoir, at flow rates of 40 ml/min or 80 ml/min [25]. The samples remained submerged by collecting leachate from the top of the column and recirculating the leachate between column and reservoir at the constant flow rate using the peristaltic pump throughout the experiment [32]. All conditions were performed in duplicate. Ferrous iron concentration and ORP were monitored on day 0, 2, 3, 5, 8, 13, 18, and 23. Day 2 onwards, the pH was adjusted to 1.75 on the same days as ferrous iron and ORP were measured. An attempt was made to conduct the experiment with a 5 % (w/v) solid concentration under the same conditions. However, the columns experienced clogging as they started to be fed with the culture on day 2.

### 2.5 Analysis methods

The pH of the cultures was determined using a pH meter (Jenway 3540). The oxidation of Fe^2+^ to Fe^3+^ is an indication of microbial growth [[26], [27], [28]], and therefore ORP was monitored using a platinum electrode with an Ag/AgCl reference electrode on monitoring days for batch (day 0, 2, 3, 5, 8, 11, 16) and column bioleaching experiments (day 0, 2, 3, 5, 8, 13, 18, 23) [29]. Leachate (2 ml) was collected and filtered using a 0.2 μm Millipore filter for further analysis. Fe^2+^ concentration was measured by titration against K_2_Cr_2_O_7_ [33]. Metal concentration in leachate was determined by inductively coupled plasma mass spectrometry (ICP-MS) as described in Tezyapar Kara et al. (2023) [26]. Metal extraction yield was calculated using the following equation (1) [34]:

(1) $$R=m/m_{0}x100$$

where R is % metal extraction, *m* is soluble metal amount in the leachate (g) and *m*_*0*_ is initial metal amount in BOS-D.

### 2.6 Statistical analyses

The descriptive statistics was performed to evaluate the differences between corresponding groups. Data were subjected to one-way ANOVA statistical method with the significance level at P < 0.01 [35,36].

## 3 Results and discussion

### 3.1 Bioleaching vs chemical leaching vs hybrid leaching

The elemental composition results showed that BOS-D primarily consisted of iron, zinc, lead, manganese, aluminium (Table 1). Autoclaved BOS-D was used to ensure bioleaching by *A. ferrooxidans* and metal leaching without interference from indigenous microorganisms [22,23]. The results showed that autoclaving BOS-D caused loss of most of the element. Although this study is not specifically centred on the effects of autoclaving, it is important to recognise that this process has the potential to modify mineral structures, affecting surface area, mineral crystallinity, and potentially leading to the creation of new mineral phases [37]. These alterations can significantly influence the efficiency of bioleaching by potentially impacting the attachment of microorganisms and the availability and accessibility of metals [38]. Therefore, further investigation is necessary to fully understand how autoclaving affects bioleaching efficiency and metal extraction. Additionally, the analysis revealed the dry matter, water content, and loss on ignition as 99 %, 1 %, and −0.19, respectively. The negative loss on ignition value, attributed to the high iron content in the materials, supports the study of Vandenberghe et al. (2010) [39] that iron oxidation might cause weight gain, surpassing the carbon-induced weight loss.

**Table 1:** Elemental composition of the autoclaved and non-autoclaved basic oxygen steelmaking dust (n = 2). The abbreviation of RSD stands for relative standard deviation.

	autoclaved		non-autoclaved	
Element	mg/kg	RSD (%)	mg/kg	RSD (%)
Y	1.3	12	1.26	2
Ce	3.8	1	4.09	8
Nd	1.8	7	1.89	12
Li	4.5	4	5.52	6
Co	10.2	3	9.73	2
Cu	126.5	1	126.5	1
Zn	18,000	8	18,950	0
Al	2660	3	2865	8
Mn	5870	4	6060	0
Fe	390,500	6	399,500	1

**Fig. 1:**
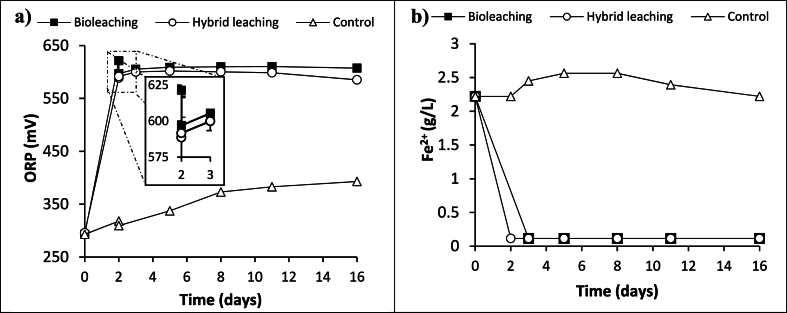
Evolution of the ORP values (mV) (a) and ferrous iron concentration (g/L) (b) of bioleaching batches overtime. As series, “bioleaching” represents the BOS-D bioleaching; “hybrid bioleaching” represents the bioleaching of residue of BOS-D from choline chloride-ethylene glycol leaching and the control is the abiotic leaching (n = 2; RSD for all series ≤20 %).

ORP values and ferrous iron consumption during bioleaching of BOS-D are shown in Fig. 1. Before inoculation (day 0), ORP value of the growth medium was around 300 mV and the ferrous iron concentration was 2.22 g/L. First, the fresh medium (ORP = 293 mV) was inoculated with adapted culture and cultivated for 2 days without BOS-D material. The ORP values of bioleaching flasks significantly increased (>575 mV compared to abiotic control (ORP = 318 mV) indicating effective bio-oxidation of Fe^2+^ to Fe^3+^ by *A. ferrooxidans* and a good bacterial growth [28,35]. When 1 % (w/v) autoclaved BOS- D was added in the flask (day 2), a sharp decrease in ORP observed (from 622 mV to 597 mV) due to alkali nature of the BOS-D (pH = 9.40). Following day (day 3) the ORP exceeded 600 mV by reaching 606 mV. This indicated that bacterial activity was continuing [26,40] (Fig. 1a). This is supported by the decrease of Fe^2+^ concentration to 0.1 g/L Fe^2+^ indicating biooxidation of ferrous iron to ferric iron by *A. ferrooxidans* (Fig. 1b). On the other hand, throughout the experiment, ORP value of the abiotic control increased slightly yet it did not exceed the 400 mV. The reason of this increase is probably due to the chemical oxidation of the Fe^2+^ in the medium by atmospheric oxygen [35].

**Fig. 2:**
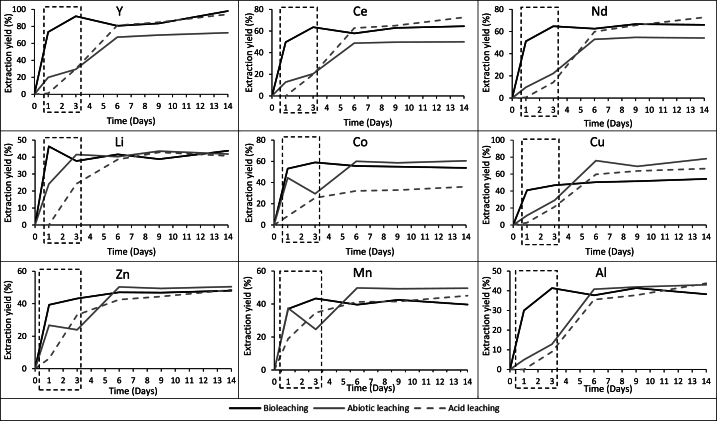
Metal extraction yields of selected elements over 14 days of leaching period (t = 0 equals to day 2 on Fig. 1, which is BOS-D addition day) (n = 2; SD for all elements ≤20 %). The dashed box encompassing data for the first three days indicated the initial leaching stage that bioleaching provided higher dissolution than controls.

Comparison of the metal extraction yields from bioleaching, abiotic leaching and acid leaching for the 9 selected elements, including Y, Ce, Nd, Li, Co, Cu, Zn, Mn and Al is shown in Fig. 2. After one day of bioleaching, 73 % Y, 50 % Ce, 51 % Nd, 46 % Li, 53 % Co, 41 % Cu, 39 % Zn, 37 % Mn, and 30 % Al were dissolved (Fig. 2). Until the end of the experiment, metal extraction yields increased between 10 % and 20 % for Y, Ce, Nd and Cu. In terms of other elements, extraction yields were slightly increased or remained stable. On the other hand, lower extraction was observed in both acid leaching and abiotic leaching compared to bioleaching on Day 3. The result indicates that biogenic Fe^3+^ contributed leaching additionally when compared to controls which can be explained by Eqs. (2), (3), (4) [12,41,42].

(2) $${2Fe}^{2+}+0.5O_{2}+{2H}^{+}\to {2Fe}^{3+}+H_{2}O$$

(3) $${Cu}^{0}+{2Fe}^{3+}\to {Cu}^{2+}+{2Fe}^{2+}$$

(4) $${Zn}^{0}+{2Fe}^{3+}\to {Zn}^{2+}{+2Fe}^{2+}$$

Abiotic leaching results were higher than acid leaching suggesting that ferrous iron acted as a reducing agent resulting effective metal extraction than acid leaching [35,51]. Day 3 onwards, metal extractions in controls followed an increasing trend until day 8. After day 8, metal extractions plateaued.

Overall, the metal extraction was always superior when bioleaching was used compared to the controls (day 1 - day 3) indicating that *A. ferrooxidans* played a key role in facilitating the extraction of the selected metals from BOD-S [9]. As a result, to save time and resource 3 days of bioleaching would be enough to extract metals from BOS-D due to extraction of all elements were nearly reached plateau.

**Fig. 3:**
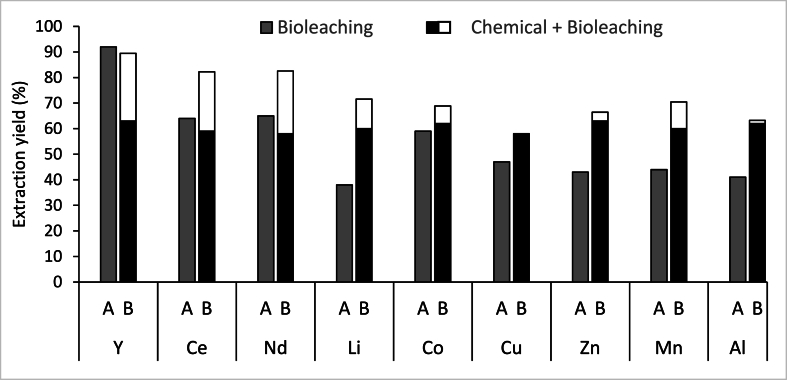
Metal extraction yields for Y, Ce, Nd, Li, Co, Cu, Zn Mn and Al at the end of the batch leaching experiments (A: Bioleaching (t = 3d); B: Hybrid leaching process = chemical leaching (t = 2h) + bioleaching (t = 14d), (n = 2; SD for all elements ≤20 %).

The comparison of the extraction efficiency between bioleaching (t = 3d), chemical leaching (t = 2h), and hybrid leaching (t = 14d) showed that bioleaching was more efficient than ChCl-EG for extraction of the rare earth elements (REEs) (Fig. 3). The hybrid leaching approach led to an additional extraction up to 27 % for REEs. For the other metals, the hybrid leaching approach resulted in an extra extraction ranging between 1 % and 12 %, except for Cu. Overall, the hybrid leaching approach resulted in the extraction of over 80 % of REEs and more than 60 % extraction of the remaining metals. Single factor ANOVA was performed to evaluate the significant differences between leaching approaches for each element (P < 0.01). Only significant difference was observed for Zn and Mn elements between bioleaching vs chemical and hybrid leaching (Fig. 3).

In Table 2, bioleaching, chemical leaching, and hybrid leaching were compared in terms of process time, extraction efficiency, and ease of implementation [10,43,44]. Chemical leaching with ChCl-EG was the most suitable method to extract critical metals, heavy metals, and others. For REEs extraction, bioleaching could be a preferable option to chemical leaching as it yielded higher extraction rates. While hybrid leaching resulted in greater metal extraction for all elements except Cu, it is seen as a less favourable method for extracting metals from BOS-D. This is because hybrid leaching introduces a more complicated process and demands additional time compared to individual processes.

**Table 2:** Comparison of the three processes, chemical leaching, bioleaching, hybrid leaching in terms of time, efficiency, and ease. The abbreviation REEs stands for rare earth elements, CRM stands for critical metals.

	Chemical leaching	Bioleaching	Hybrid leaching
REEs	+ +	+ + +	+
CRM	+ + +	+ +	+
Heavy metal and others	+ + +	+ +	+

### 3.2 Scale-up bioleaching

**Fig. 4:**
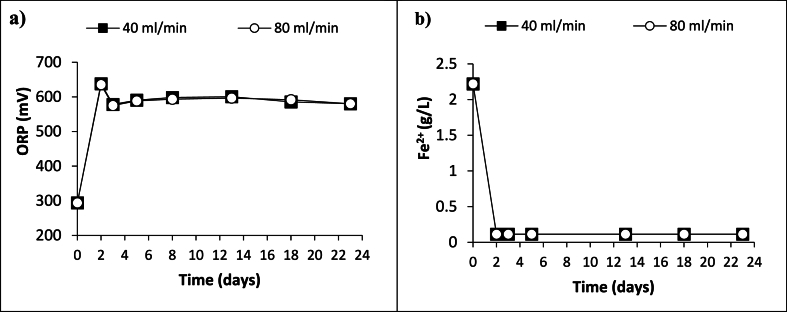
Evolution of the ORP values (mV) (a) and ferrous iron concentration (g/L) (b) during column experiments (n = 2; RSD for all series ≤20 %).

Evolution of ORP values and ferrous iron concentrations during column bioleaching experiments were shown in Fig. 4. As expected, ORP values of the culture increased in the first two days (Fig. 4a). Supportively, ferrous iron was consumed by *A. ferrooxidans* (Fig. 4b) as observed in the bioleaching batches. Then, on day 2, columns were started to feed with the culture from the bottom with different recirculation rates, 40 ml/min and 80 ml/min. Compared to the agitation condition (Fig. 1a), the decrease in the ORP was slower but still occurred (Fig. 4a). On the following days, the ORP slightly increased. No significant difference in the ORP values and the ferrous iron concentration was observed between different recirculation rates.

**Fig. 5:**
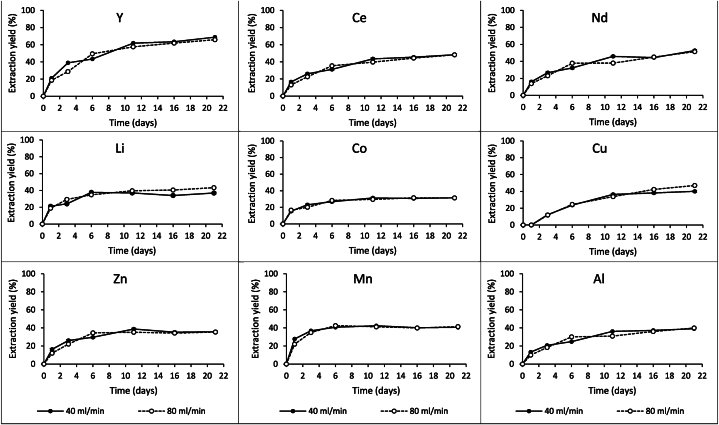
Extraction yields of Y, Ce, Nd, Li, Co, Cu, Zn Mn and Al with different recirculation rates of bioleaching process (t = 0 equals to day 2 in Fig. 4, which the beginning of the circulation) (n = 2; SD for all elements ≤20 %).

Fig. 5 shows the extraction of Y, Ce, Nd, Li, Co, Cu, Zn, Mn, and Al with different recirculation rates over 21 days of column scale bioleaching experiments. After one day of bioleaching metal extraction between 10 % and 28 % was observed for all elements except Cu. Until Day 6 an increasing extraction was observed for all metals. Day 11 onwards, extraction reached a plateau for most of the elements except REEs. Extraction of REEs continued to increase until the end of the experiment. There was no significant difference between 40 ml/min and 80 ml/min recirculation rates. When it compared to batch scale leaching results, a decrease in the extraction yields of most metals was observed when the bioleaching scaled up, except for a slight increase in Mn and Al. This may have been caused by heat lost and insufficient solid-liquid-gas interaction in the column [12]. More controlled environment is needed to use percolation method as an industrial application. Fine particles caused blockage when more than 1 % solid concentration was used. However, this solid concentration is too low for the process to be economical. Permeability is a critical factor for the for industrial scale leaching operations where the percolation method (heap or vat) was used as poor permeability cause channelling and a decrease in metal extraction rate [49]. Agglomeration is one of the techniques that is used in mineral processing to enhance the permeability [12,48]. Agglomeration, where Portland cement, lime or vinyl polymers are used as an agglomeration binder, can potentially be used to increase the permeability in metallurgical waste leaching [50]. In addition, Yılmaz et al. (2020) [50] suggested nut shell addition in the heap as a percolation enhancer. They found that nut shell addition markedly increased the permeability of the ore and hence the leach efficiency for gold recovery from the gold ore heaps. In addition, we have tested the flow recirculation in the column at 20 % (w/v, 200 g BOS-D in the column) solid concentration using DI water. After one day flow test we have not experienced clogging. However, we when we run the column with *A. ferrooxidans* culture we experienced clogging. That can because of the difference between density or the viscosity of the culture and DI water or the presence of jarosite precipitates in the *A. ferrooxidans* culture. We have tried to run the bioleaching columns in two different occasions and experienced clogging above 1 % (w/v) solid concentration. However, exploring the reason of this was beyond the scope of this study. In addition, optimisation of air flow may enhance the metal extraction yield [45]. Alternatively, stirred tank reactors can be considered as a scale up alternative due to it provides enhanced gas distribution and solid-liquid interaction [9,46]. However, this would be an expensive option when it compared to the percolation method (heap or vat).

The amounts of extracted metals from the bioleaching column system, with a recirculation rate of 40 ml/min, at day 11 and day 21 are presented in Table 3. On day 11, 3.3 mg/kg REEs, 51 mg/kg critical metals and 10,536 mg heavy metals and other metals were extracted. Extracted metal amount were slightly increased for the REEs (up to 7 %) until the end of the experiment resulted 3.7 mg/kg REEs extraction (Day 21). At the end of the experiment 69 % Y, 48 % Ce, 53 % Nd, 37 % Li, 32 % Co, 40 % Cu, 36 % Zn, 39 % Al and 41 % Mn dissolved which corresponds to 0.89 mg/kg Y, 1.86 mg/kg Ce, 0.95 mg/kg Nd, 2 mg/kg Li, 3 mg/kg Co, 51 mg/kg Cu, 6555 mg/kg Zn, 1040 mg/kg Al, 2385 mg/kg Mn. A total of 10,039 mg/kg elements were extracted from BOS-D at the end of the column experiment using 40 ml/min recirculation rate, while it was higher on day 11 (10,590 mg/kg). As a result, to save time and resource, operating the bioleaching column system for 11 days would be enough to extract metals from BOS-D, considering that the extraction of the most abundant elements had nearly plateaued.

**Table 3:** Comparison of the extracted amounts of Y, Ce, Nd, Li, Co, Cu, Zn, Mn and Al on day 11 and day 21 (t = 0 equals to day 2 in Fig. 4, which the beginning of the recirculation) in column scale bioleaching experiments using 40 ml/min recirculation rate. The abbreviation REEs stands for rare earth elements, CRM stands for critical metals.

Element	Initial metal concentration (mg/kg)	Day 11		Day 21	
		Metal extraction yield (%)	Amounts of recovered element (mg/kg)	Metal extraction yield (%)	Amounts of recovered element (mg/kg)
**REE**					
Y	1.3	62	0.80	69	0.89
Ce	3.8	43	1.67	48	1.86
Nd	1.8	46	0.83	53	0.95
**Total**	**7.0**	**-**	**3.3**	**-**	**3.7**
**CRM**					
Li	4.5	37	1.7	37	1.7
Co	10.2	31	3.2	32	3.2
Cu	126.5	36	46.0	40	50.7
**Total**	**141**	**-**	**51**	–	**56**
**Heavy metal and others**					
Zn	18,300	39	7089	36	6555
Al	2660	36	962	39	1040
Mn	5870	42	2485	41	2385
**Total**	**26,830**	**-**	**10,536**	**-**	**9980**
**Total elements**	**26,978**	**-**	**10,590**	**-**	**10,039**

The amounts of extracted metals from the bioleaching column system, with a recirculation rate of 40 ml/min, at day 11 and day 21 are presented in Table 3. On day 11, 3.3 mg/kg REEs, 51 mg/kg critical metals and 10,536 mg heavy metals and other metals were extracted. Extracted metal amount were slightly increased for the REEs (up to 7 %) until the end of the experiment resulted 3.7 mg/kg REEs extraction (Day 21). At the end of the experiment 69 % Y, 48 % Ce, 53 % Nd, 37 % Li, 32 % Co, 40 % Cu, 36 % Zn, 39 % Al and 41 % Mn dissolved which corresponds to 0.89 mg/kg Y, 1.86 mg/kg Ce, 0.95 mg/kg Nd, 2 mg/kg Li, 3 mg/kg Co, 51 mg/kg Cu, 6555 mg/kg Zn, 1040 mg/kg Al, 2385 mg/kg Mn. A total of 10,039 mg/kg elements were extracted from BOS-D at the end of the column experiment using 40 ml/min recirculation rate, while it was higher on day 11 (10,590 mg/kg). As a result, to save time and resource, operating the bioleaching column system for 11 days would be enough to extract metals from BOS-D, considering that the extraction of the most abundant elements had nearly plateaued.

Moreover, techno-economic assessment (TEA) of bioleaching of BOS-D was conducted [55]. Although TEA is not a focus of this research, it is important to demonstrate how the experimental results can be implemented and benefit from. Briefly, TEA evaluated two distinct industrial scale bioleaching technologies, including an aerated bioreactor and an aerated and stirred bioreactor, for various recovery scenarios. The extraction yields from previous batch experiments [26], were used to calculate the revenue from aerated and stirred bioreactor. On the other hand, results that obtained from scale up experiment on day 11 were used to derive the extraction yield for the aerated bioreactor plant where for each element the extraction data was proportionally adjusted to compute the revenue [55]. As a result, bioleaching of BOS-D for Zn recovery revealed no estimated profit within the 19 years after the project started for the aerated and stirred bioreactor plant. However, there was a potential for the plant to become profitable beyond this initial phase. Despite the limitations, the TEA emphasized the significance of selective metal recovery and plant design, and underscored the major expenses associated with the process.

## 4 Conclusion

This study uncovers significant findings and practical implications regarding the extraction of metals from basic oxygen steelmaking dust (BOS-D). Notably, during the initial leaching stage, bioleaching conducted in batch outperformed control methods, demonstrating its efficacy in extracting various metals. Bioleaching further exhibited promising results with the extraction of REEs and other metals, outperforming the alternatives in certain cases. Chemical leaching, while efficient, proved to be less suitable for REEs extraction. The hybrid leaching approach, combining a deep eutectic solvent and bioleaching, yielded additional REEs extraction. Scaling up the bioleaching process highlighted challenges, with decreased extraction rates observed for most metals. The study findings suggest that for optimal efficiency and resource use, concluding column bioleaching after 11 days is advisable. Furthermore, the research emphasizes the need for controlled environments when implementing percolation methods at an industrial scale. As fine particles cause blockage at high solid concentration (>1 %) for BOS-D increasing the permeability using agglomeration or percolation enhancer can be suggested for high solid concentrations to make the process economical. Alternatively, a stirred tank reactor is proposed as a scalable solution for the industrial application of bioleaching, though further investigation is required to optimise aeration and agitation rates. However, stirred reactor would be more expensive when it compared to percolation method. Thus, future research should focus on optimising these proposed solutions. One key area is enhancing permeability by developing and testing agglomeration techniques or percolation enhancers for high solid concentrations in BOS-D. Another important direction is optimising stirred tank reactors, specifically by investigating the optimal aeration and agitation rates to improve their efficiency and cost-effectiveness for bioleaching. Additionally, further exploration of hybrid leaching processes, particularly the integration of deep eutectic solvents with bioleaching, could maximize the extraction of REEs and other valuable metals. Finally, it is crucial to continue refining techno-economic assessments to better understand the economic viability and scalability of these bioleaching. Overall, this study not only advances our understanding of bioleaching applications for secondary resources but also provides valuable insights into the limitations and opportunities for scaling up this eco-friendly metal extraction process. These findings can inform and guide future research on sustainable metal recovery from industrial waste materials.

## Data availability statement

All the data supporting this study are included within the article.

## CRediT authorship contribution statement

**Ipek Tezyapar Kara:** Writing – original draft, Methodology, Investigation, Formal analysis, Conceptualization. **Victoria E. Huntington:** Methodology, Investigation, Formal analysis. **Nuannat Simmons:** Validation, Methodology, Formal analysis, Data curation. **Stuart T. Wagland:** Writing – review & editing, Validation, Supervision, Resources. **Frederic Coulon:** Writing – review & editing, Validation, Supervision, Resources, Project administration, Methodology, Funding acquisition, Conceptualization.

## Declaration of competing interest

The authors declare the following financial interests/personal relationships which may be considered as potential competing interests: Frederic Coulon is a co-Editor of the Environment Section.
